# Construction of a simple biocatalyst using psychrophilic bacterial cells and its application for efficient 3-hydroxypropionaldehyde production from glycerol

**DOI:** 10.1186/2191-0855-3-69

**Published:** 2013-12-05

**Authors:** Takahisa Tajima, Koji Fuki, Naoya Kataoka, Daizou Kudou, Yutaka Nakashimada, Junichi Kato

**Affiliations:** 1 Department of Molecular Biotechnology, Graduate School of Advanced Sciences of Matter, Hiroshima University, 1-3-1 Kagamiyama, Higashi-Hiroshima, Hiroshima 739-8530, Japan

**Keywords:** Biocatalysis, Psychrophilic bacterium, 3-hydroxypropionaldehyde, Biomass

## Abstract

Most whole cell biocatalysts have some problems with yields and productivities because of various metabolites produced as byproducts and limitations of substrate uptake. We propose a psychrophile-based simple biocatalyst for efficient bio-production using mesophilic enzymes expressed in psychrophilic *Shewanella livingstonensis* Ac10 cells whose basic metabolism was inactivated by heat treatment. The 45°C heat-treated cells expressing *lacZ* showed maximum beta-galactosidase activity as well as chloroform/SDS-treated cells to increase membrane permeability. The fluorescent dye 5-cyano-2,3-ditolyl-tetrazolium chloride staining indicated that most basic metabolism of Ac10 was lost by heat treatment at 45˚C for 10 min. The simple biocatalyst was applied for 3-HPA production by using *Klebsiella pneumoniae dhaB* genes. 3-HPA was stoichiometrically produced with the complete consumption of glycerol at a high production rate of 8.85 mmol 3-HPA/g dry cell/h. The amount of 3-HPA production increased by increasing the concentrations of biocatalyst and glycerol. Furthermore, it could convert biodiesel-derived crude glycerol to 3-HPA.

## Introduction

Microorganisms are used as platform cell factories to produce various building block chemicals from renewable resources. The development of efficient microbial strains producing target compounds at high titers, yields, and productivities is required for industrial production. Metabolic engineering combined with systems biology has contributed to the enhanced production of chemicals and materials by redistributing and optimizing metabolic fluxes (Lee et al. 2011); however, this approach is often extremely laborious, costly, and difficult (Hodgman and Jewett 2012; Kwok 2010). On the other hand, thermostable enzymes expressed in *Escherichia coli* have achieved efficient bio-conversions with nearly 100% yields (Iwamoto et al. 2007; Ye et al. 2012). The process in the preparation of biocatalysts is easy and simple, which is briefly described as follows: thermostable enzymes are expressed in *E. coli* for designing synthetic pathways of chemicals production; the resulting cells are treated by heat typically at 70 C for 30 min to inactivate the *E. coli* basic metabolism; finally the *in vitro* metabolic pathway is constructed to produce target compounds only by the activities of thermostable enzymes in the cells. The biocatalysts have succeeded in the efficient bio-conversions of lactate or malate from glucose with high yields because of the increase of the flux in the bio-conversion pathway and the membrane permeability of substrate by heat treatment. This biocatalyst is called *E. coli*-based “simple biocatalyst”.

We considered the construction of a simple biocatalyst using psychrophilic bacterial cells (psychrophile-based simple biocatalyst) instead of *E. coli* cells. This biocatalyst has significant advantages in the use of abundant mesophilic enzymes for designing bio-conversion pathways, in addition to thermostable enzymes because the use of psychrophilic hosts allows the heat treatment temperature for inactivation of basic metabolisms to be lower than that of *E. coli* based simple biocatalysts. Out of 2075 “completed and published” genome sequences in genome information database GOLD (http://www.genomesonline.org/; June, 7th, 2013), most genome sequences (1799 genomes; 86.7%) belong to mesophilic organisms, while those in “hyperthermophile,” “thermophile,” or “thermotolerant” consist of only 232 genomes (11.2%). Psychrophile-based simple biocatalysts may be able to exploit nearly all enzymes to produce chemicals. Furthermore, decreasing the heat treatment temperature to an ordinary temperature limits concerns about the excess disruption of the cellular structure and thermal decomposition of coenzymes, such as NADP^+^ and NADPH, which are problems in *E. coli*-based simple biocatalysts (Ye et al. 2013).

In this study, the psychrophilic bacterium *Shewanella livingstonensis* Ac10 was selected as a host for the simple biocatalyst. Ac10 isolated from Antarctic sea shows optimum growth at 18˚C but cannot grow above 30˚C (Miyake et al. 2007). A gene expression system using the broad-host-range plasmid RSF1010 and its derivatives are available in Ac10, and proteins expressed at low temperatures have been revealed by proteomics analysis (Kawamoto et al. 2007; Kawamoto et al. 2009). We constructed the innovative psychrophile-based simple biocatalyst for efficient bio-conversion by recombinant mesophilic enzymes expressed in a psychrophilic bacterium *S. livingstonensis* Ac10 whose basic metabolism was inactivated by heat treatment. We evaluated the activity of recombinant *E. coli* β-galactosidase expressed in *S. livingstonensis* Ac10 and basic metabolism of Ac10 when cells were treated by heat, and applied it to the simple biocatalyst for 3-HPA production from glycerol by using *Klebsiella pneumoniae dhaB* genes. Although the conversion from glycerol to 3-HPA is not multistep enzymatic reaction (pathway) but single step reaction, psychrophile-based simple biocatalyst will be applicable to *in vitro* metabolic pathway if it succeeds in efficient production.

## Materials and methods

### Cell strains and growth conditions

The bacterial strains and plasmids used in this study are described in Table 1. All *E. coli* strains were grown in Luria-Bertani liquid or solid agar medium at 37°C with 100 mg/L ampicillin when appropriate. *S. livingstonensis* Ac10-Rif^r^ (kindly provided by Prof. T. Kurihara in Kyoto University; Kawamoto et al. 2009) was grown with triptic soy broth (TSB; Difco Laboratories, Detroit, MI, USA) at 18°C with 50 mg/L rifampicin. *E. coli* DH5*α* (TOYOBO, Japan) was used for the construction of the recombinant plasmids. Resulting plasmids were introduced into *S. livingstonensis* by transconjugation using *E. coli* S17-1 (Kawamoto et al. 2009). Transformed *S. livingstrensis* cells were selected using rifampicin and ampicillin at concentrations of 50 mg/L and 100 mg/L, respectively. To overexpress *K. pneumoniae* glycerol dehydratase (DhaB) in *S. livingstonensis* Ac10-Rif^r^, *dhaB* genes were cloned into the vector pHA12 and expressed in Ac10-Rif^r^ by 100 μM IPTG induction. To prepare Ac10/pHA12-dhaB cells for a resting cell reaction, Ac10-Rif^r^/pHA12-dhaB cells grown overnight in TSB medium were inoculated into 100 mL of TSB culture medium (1% inoculum). Cultures were incubated in Erlenmeyer-flasks under constant agitation on a rotary shaker, maintained at 120 rpm. Cell growth was recorded by measuring the optical density of the culture broth at 660 nm (OD_660_) and the dried weight of the cells. For the viability test of heat-treated cells, diluted samples were plated on TSB ager and cultured at 18°C. After 3–5 days the colonies were qualitatively observed.

**Table 1:** Bacterial strains and plasmids used in this study

**Strains or plasmids**	**Relevant characteristics**	**Source or reference**
Strains		
*E. coli* strains		
DH5α	Cloning host *supE*44 Δ*lac*U169 (*Φ*80 *lac*Z ΔM15) *hsdR* 17 *rec*A1 *end*A1 *gyrA*96 *thi*-1 *relA*1	TOYOBO
S17-1	*rec*A *pro hsdR* RP4-2-Tc::Mu-Km::Tn*7*	Simon et al. (1983)
*S. livingstonensis* strains		
Ac10-Rif^r^	Parent strain, rifampin resistant mutant of Ac10	Kawamoto et al. (2007)
Ac10-Rif^r^/pHA10-lacZ	Ac10-Rif^r^ harboring pHA10-lacZ	This study
Ac10-Rif^r^/pHA12-dhaB	Ac10-Rif^r^ harboring pHA12-dhaB	This study
*K. pneumoniae* T7	1,3-propanediol producing strain	Laboratory’s stock
Plasmids		
pHA10	Amp^r^, expression vector	Arai et al. (1991)
pHA12	Amp^r^, expression vector	Arai et al. (1991)
pQF50	Amp^r^, broad-host-range vector	Farinha and Kropinski (1990)
pHA10-lacZ	Amp^r^, pHA10 containing *lacZ*	This study
pHA12-dhaB	Amp^r^, pHA12 containing *K. pneumoniae dhaB*	This study

### Plasmid construction

Standard procedures for plasmid DNA preparation, restriction enzyme digestion, ligation, and transformation were used (Sambrook 1989). PCR reactions were performed with KOD plus Neo (Toyobo, Ohtsu, Japan) according to the manufacturer’s recommendations. The *E. coli lacZ* gene was amplified from pQF50 (Farinha and Kropinski 1990) by using primers lacZ_F (5′-AGAGGGTATTAATAATGAAAGGGAA-3′) and lacZ_R (5′-CCCAAGCTTTGCCCGGTTATTATTATTTTTGA-3′). The PCR product was cloned between the *Sma*I and *Hind*III site of broad-host range expression vector pHA10 (Arai et al., 1991). The *dhaB1*, *dhaB2*, and *dhaB3* genes (accession number AB859215) were amplified with ribosome binding site (GGAGA) from *K. pneumoniae* T7 genomic DNA using primers dhaB_F (5′-ATGCGAATTCGGAGAGATGAACAATGAAAAGATCAAAACGA-3′) and dhaB_R (5′-ATGCGGTACCTTAGCTTCCTTTACGCAGCT -3′). The PCR fragment was ligated with *EcoR*I-*Kpn*I digested pHA12 (Arai et al. 1991) to construct pHA12-dhaB.

### β-galactosidase activity

For β-galactosidase assays, cells were harvested from aliquots of 1 mL of 24-h cultures of *S. livingstonenesis* (OD_660_ = 0.5-1.0) by centrifugation and were washed twice with 0.9% NaCl. Bacterial pellets were resuspended in Z-buffer (36 mM NaH_2_PO_4_, 67 mM Na_2_HPO_4_, 0.1 mM MgCl_2_, 2 mM MgSO_4_, 2.7 mL/l β-mercaptoethanol) before lysis with 20 μL of chloroform and 10 μL of 0.1% SDS by a Pasteur pipet or heat treatment. β-Galactosidase assays were performed at 37°C using the Miller protocol (Miller 1972) with modification. Briefly, 200 μL of 4 mg/mL ONPG (Nacalai tesque, Kyoto, Japan) was added into 500 μL of cell suspension, and the mixtures were incubated at 20°C. After a 10-min incubation, 250 μL of stop buffer (1 M Na_2_CO_3_) was added, and the absorbance measured at 420 nm and 550 nm. β-Galactosidase activities [Miller units (nmoles/minute)] were calculated using the following formula (Miller 1972): β-Galactosidase activity (Miller units) = (OD_420_ -1.75 × OD_550_)/(t × V × OD_660_) × (1/0.0045) × 1000, where t = length of incubation (min) and V = volume of culture used in the assay (μL).

### CTC staining assay

Cell viability was evaluated by CTC staining assay. A CTC (5-Cyano-2,3-ditolyl tetrazolium chloride) rapid staining kit for microscopy (Dojindo Laboratories, Kumamoto, Japan) was used for cell respiratory detection. CTC working solutions (final conc.: 50 mM) were prepared by dissolving CTC in 750 μL sterilized distilled water. Triplicate 1-mL samples treated with 20 μL of CTC solution and 5 μL enhancing reagent B were incubated for 30 min at 18°C. Stained cells were observed by inverted microscopy (E600 Eclipse, Nikon, Tokyo, Japan) equipped with blue excitation and red emission filters.

### 3-HPA production

Cultivated cells were collected by centrifugation (4°C, 5 min, 3220 × *g*) and washed twice with 70 mM potassium phosphate buffer (pH 8.0). Reactions were started by the addition of glycerol, vitamin B_12_ (1.5 μM in final concentration; Sigma-Aldrich Japan, Tokyo, Japan), and 50 mM KCl into the equal parts of the cell suspension. The reactions were carried out at 37°C and stopped by the addition of chloroform. Supernatant was obtained by centrifugation of the reaction mixture.

### Analytics

Quantification of 3-hydroxypropionaldehyde (3-HPA) was based on the modified colorimetric method of Circle *et al.* (Circle et al. 1945). Briefly, 150 μL of 10 mM tryptophan dissolved in 0.05 N HCl was mixed with 200 μL of sample. After addition of 600 μL of 37% HCl, the mixture was incubated at 37°C for 20 min, and the absorbance was meadured at 560 nm. Acrolein (0–1 mM) was used for obtaining the standard curve. Glycerol was measured by a high performance liquid chromatograph system equipped with a UV detector and an ion exclusion column (RSpak KC-811, 8.0 mm ID × 300 mm L; Shodex, Tokyo, Japan). The column was eluted at 60°C using 0.1% (v/v) phosphate as a mobile phase at a flow rate of 0.7 mL/min.

## Results

### Heat treatment condition for simple biocatalyst

**Figure 1:**
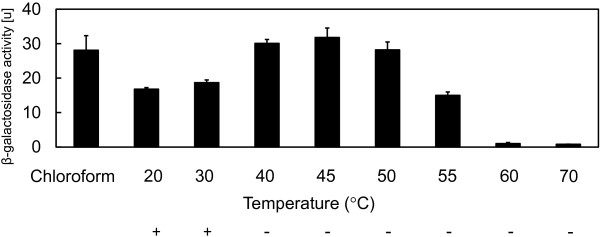
**β-Galactosidase activities by the cells treated with chloroform and heat treatment at 20–70°C for 10 min.** Error bars represent standard deviation of the mean (*n* = 3). Viabilities of heat-treated cells are indicated as “+” and “-“.

**Figure 2:**
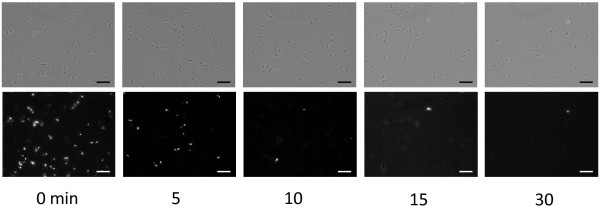
**CTC staining cells heat-treated at 45°C for 0, 5, 10, 15, and 30 min.** Phase-contrast micrographs (upper photos) and fluorescence micrographs (lower photos) of the same fields are shown. Bars represent 10 μm.

To evaluate the effect of heat treatment on *S. livingstonenesis* Ac10, we measured the β-galactosidase activity of the recombinant Ac10 cells in which the *E. coli lacZ* gene was expressed under the control of a *tac* promoter. Ac10-Rif^r^/pHA10-lacZ cells were pretreated with heat at 20–70°C for 10 min. The cells lost viability when treated over 30°C (Figure 1). The β-galactosidase activity at 20°C in the heat-treated cells or chloroform/SDS-treated cells was measured by a standard protocol (Figure 1). Heat treatment at 20-45°C resulted in an increase in the β-galactosidase activity along with the increase of treatment temperature. The highest activity was obtained in the cells with heat treatment at 45°C as well as those with chloroform/SDS treatment which is generally used to permeabilize the cells. The supernatant suspension of cells heat-treated at 45°C had 14% residual β-galactosidase activity. On the other hand, cells heat-treated above 45°C showed decreased β-galactosidase activity with the increase of temperature, and this activity was almost lost at 60°C and 70°C. We carried out the CTC staining of cells treated for 5, 10, 15, and 30 min at 45°C to determine heat treatment time for the inactivation of basic metabolism in Ac10-Rif^r^ cells. CTC was converted to the insoluble fluorescent dye CTC-formazan when CTC was reduced as an artificial redox partner instead of the final electron acceptor oxygen by respiring bacteria (Bovill et al. 1994). After CTC-staining, the 5-min heat-treated cells showed fluorescence with half intensity compared with that of non-treated reference cells (Figure 2). Fluorescence was scarcely detected in nearly all cells treated for more than 10 min.

### 3-HPA production by simple biocatalyst

**Figure 3:**
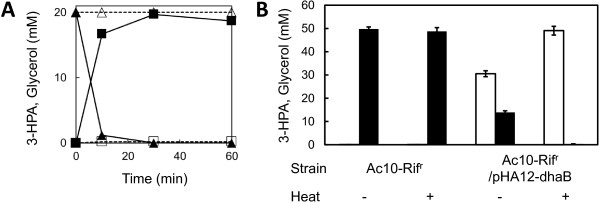
**3-HPA production by simple biocatalyst. (A)** Time course of 3-HPA production (squares) and glycerol consumption (triangles) from 20 mM glycerol in the reaction by Ac10-Rif^r^ (dashed line, open marker) and Ac10-Rif^r^/pHA12-dhaB (solid line, filled marker). **(B)** Concentration of 3-HPA (light) and glycerol (black) in the reaction by Ac10-Rif^r^ and Ac10-Rif^r^/pHA12-dhaB cells with (+) or without (-) heat treatment. Error bars represent standard deviation of the mean (*n* = 3).

To apply the Ac10 simple biocatalyst to 3-HPA bio-production, *dhaB* gene cluster containing *dhaB1*, *dhaB2*, and *dhaB3* genes from *K. pneumoniae* T7 was cloned into a broad-host-range plasmid. Their amino acid sequences have 100% identities to corresponding genes in *K. pneumoniae* MGH 78578. Glycerol dehydratase, the gene product of *dhaB*, catalyzes conversion of glycerol to 3-HPA. At first, we failed to obtain any recombinant *E. coli* when the *dhaB* cluster was introduced into *lacI*^q^-less pHA10 plasmid containing a constitutive *tac* promoter. It seemed to be the result of the toxic effect of *dhaB* overexpression on *E. coli* cells. Therefore, we used pHA12 (Arai et al. 1991) that harbors *lacI*^q^ and its *tac* promoter controllable by the Lac repressor and isopropyl-β-D-thiogalactopyranoside (IPTG) addition. By using pHA12, we succeeded the construction of the recombinant plasmid pHA12-dhaB for overexpression of the *dhaB* cluster. A transformant (Ac10-Rif^r^/pHA12-dhaB) was obtained by introducing pHA12-dhaB into Ac10-Rif^r^. After Ac10-Rif^r^/pHA12-dhaB cells were treated by heat (at 45°C and for 15 min), 3-HPA production was performed in phosphate buffer (pH 8.0) at 37°C by the addition of 20 mM glycerol, 10 μM vitamin B_12_ and 50 mM KCl (final concentration) to cell suspension. 3-HPA was stoichiometrically produced with the complete consumption of glycerol within 30 min by Ac10-Rif^r^/pHA12-dhaB cells (5.57 g dry cell/L), while both glycerol consumption and 3-HPA production were not observed when the parent strain (Ac10-Rif^r^) was used for the reaction (Figure 3A). Heat-treated Ac10-Rif^r^/pHA12-dhaB could completely convert 50 mM glycerol to 3-HPA in 1 h; however, the cells without heat treatment partially consumed glycerol (35 mM) and produced only 31 mM 3-HPA (Figure 3B). The amount of 3-HPA produced increased with increasing concentrations of heat-treated cells and glycerol. 3-HPA production of 28.0 g dry cell/L from 300 mM glycerol reached a high titer (223 ± 10.3 mM) with high productivity (7.95 mmol/g dry cell/h). Although almost the same amount of 3-HPA (225 ± 6.5 mM) was produced from 400 mM glycerol, the yield and productivity were decreased to 0.56 and 4.80, respectively (Table 2).

**Table 2:** **Production of 3-HPA from glycerol comparing to resting cell reactions of *L. reuteri***

**Strain**	**Dry cell weight (g/L)**	**Glycerol (mM)**	**3-HPA (mM)**	**Yield**	**Productivity (mmol/g dry cell/h)**	**Reference**
*L. reuteri* ATCC53608	40-60	200	160	0.80	4.27	Doleyres et al. 2005
	(4.5 × 10^10^	400	235	0.59	6.27	
	×cfu/mL)					
*L. reuteri* CG001	11.1	200	82	0.41	3.69	Chen et al. 2011
	25.3	200	195	0.98	7.71	
*S. livingstonenesis* Ac10-Rif^r^/pHA12-dhaB	5.57	50	49.3 ± 1.9	0.99	8.85	This study
	9.48	200	82.9 ± 1.0	0.41	8.74	
	19.52	200	142 ± 1.7	0.71	7.27	
	28.03	300	223 ± 10.3	0.74	7.95	
	46.86	400	225 ± 6.5	0.56	4.80	

Finally, we examined the simple biocatalyst for the ability to produce 3-HPA from crude glycerol. The crude glycerol used was waste derived from biodiesel production by an alkaline process in Thailand and consisted of 57.9% (w/w) glycerol, 30.2% methanol, 12.5% fatty acids, and 2.7% ash. In the reaction mixture containing 64.3 mM glycerol derived from a crude glycerol sample, Ac10-Rif^r^/pHA12-dhaB (45.7 g dry cell/L) could convert this to 63.5 ± 5.96 mM 3-HPA in 1 h.

## Discussion

In this study we constructed a simple biocatalyst for the production of value-added chemicals by using heat-treated psychrophilic cells expressing mesophilic enzymes. The psychrophile-based simple biocatalyst showed that the membrane permeability of the substrate was increased by heat treatment at 45°C and most of the recombinant enzymes remained in the cells. Together with the CTC staining, heat treatment at 45°C for 10 min is adequate to inactivate the basic metabolism of Ac10. Thus, a psychrophilic-based simple biocatalyst can be constructed by using mostly mesophilic enzymes without inactivation by heat treatment. Therefore, we concluded that mesophilic enzymes can be used for a psychrophilic-based simple biocatalyst, and we could apply it to 3-HPA production from glycerol.

3-HPA is a valuable compound used as a precursor for 1,3-propanediol (1,3-PDO), 3-propionic acid, and acrylic acid, which are useful for polymer production. 3-HPA is produced from glycerol by mesophilic bacteria, such as *Lactobacullus reuteri*, *K. pneumoniae*, *Clostridium butyricum*, *Enterobacter agglomerans*, and *Citrobacter freudii* (Vollenweider and Lacroix 2004). Some studies have reported the biological production of 3-HPA by extracted enzymes (Slininger et al. 1983) and whole cells of *K. pneumoniae* (Slininger and Bothast 1985) and *E. agglomerans* (Barbirato et al. 1996). However, 3-HPA is an intracellular intermediate and quickly reduced to 1,3-PDO by a NADH-linked 1,3-PDO dehydrogenase in most 1,3-PDO native producers except for *L. reuteri* and some *Lactobacillus* strains, which are only the isolates shown to accumulate 3-HPA extracellularly. Barbirato *et al*. ( 1996) reported that 1,3-PDO native producers, such as *K. pneumoniae* and *E. agglomerans,* accumulated 17 to 30 mM of 3-HPA into the cells and the yield of 3-HPA production was very low (<0.04). Enzymatic 3-HPA production by glycerol dehydratase extracted from *Lactobacillus* sp. produced 95.8 mM 3-HPA with consumption of 97.7 mM glycerol (Slininger et al. 1983) and the yield was much higher (0.98). However, extractions of enzymes were costly and had handling difficulties for industrial applications. The simple biocatalyst is not only easy to be prepared and but also effective for 3-HPA production with high yield (0.99) and high productivity (8.85 mmol/g dry cell/h) (Table 2).

The productivity was still higher than any previously reported in the resting cell reaction of *L. reuteri* ATCC53608 (Doleyres et al. 2005) and its derivative mutant CG001 acclimatized in 3-HPA-containing medium (Chen et al. 2011) (Table 2), which was the only species shown to sustain large amounts of 3-HPA produced from glycerol (Vollenweider and Lacroix 2004). The titer of 3-HPA by our simple biocatalyst was almost the same as those by *L. reuteri*. We conducted the simple biocatalyst reaction at a cell density of 46.9 g dry cell/L and 400 mM glycerol, but only 225 mM 3-HPA was produced. It is probably a result of the inhibitory effects of glycerol and 3-HPA on glycerol dehydratase (Doleyres et al. 2005). Glycerol deactivates glycerol dehydratase by the homolysis of the coenzyme upon binding to the apoenzyme (Toraya 2000). It can be overcome by ATP-dependent reactivation factors in 1,3-PDO native producers (Toraya 2000). Although the simple biocatalyst did not have the reactivating system, 3-HPA yields in the reaction from high glycerol concentrations were only slightly lower than those of the *L. reuteri* strains (Table 2). Another type of DhaB in *Clostridium butyricum* (Raynaud et al. 2003), which does not require coenzyme vitamin B_12_ for its activity, may be an alternative for solution of the inhibitory effect by high concentrations of glycerol. Carbohydrazide can scavenge aqueous 3-HPA by forming hydrazone and its use was proposed to overcome the inhibitory effect of 3-HPA and to produce higher amounts of 3-HPA (Krauter et al. 2012). Although very high accumulation of 3-HPA scavenger-adduct (2 M) in the medium was achieved by using a carbohydrazide scavenger, no methods are available now to recover 3-HPA from scavenger adducts. To apply the simple biocatalyst to industrial production of 3-HPA, measures for high cell-density immobilization and protection from toxic 3-HPA should be established.

The result of 3-HPA conversion from crude glycerol suggested that impurities in the crude glycerol sample did not affect the bio-conversion rate of the simple biocatalyst. The inhibitory effects of crude glycerol have been reported on cell growth (Venkataramanan et al. 2012) and cell viability (Gonzalez-Pajuelo et al. 2004), which resulted in decreased production of 1,3-PDO compared with that from pure glycerol. Because *S. livingstonensis* Ac10 cells are dead after heat treatment, the inhibitory effects of crude glycerol on “cell growth” and “cell viability” should not be of concern in the case of the simple biocatalyst, and it is one of its advantages. Taken together, the psychrophile-based simple biocatalyst is a useful and effective option for bio-production of valuable chemicals. For further improvement, the system for regeneration of cofactors, such as NAD (P) H and ATP, should be developed.

## Competing interests

The authors declare that they have no competing interests.
